# Radiation dose optimization in coronary CT angiography clinical application of absolute phase gating in patients with atrial fibrillation

**DOI:** 10.1002/acm2.70666

**Published:** 2026-06-23

**Authors:** Yuyang Li

**Affiliations:** ^1^ Department of Radiology Wuhan Fourth Hospital Wuhan Hubei China

**Keywords:** absolute phase gating, atrial fibrillation, coronary CT angiography, diagnostic accuracy, dose optimization, image wisely, radiation dose

## Abstract

**Background:**

Atrial fibrillation (AF) poses significant challenges for coronary CT angiography (CCTA) due to R‐R interval variability, often necessitating high radiation dose protocols. Absolute phase gating, which uses fixed millisecond delays rather than percentage‐based cardiac cycle timing, may optimize image quality while reducing radiation exposure.

**Purpose:**

To evaluate whether absolute phase gating reduces radiation dose while maintaining diagnostic accuracy and image quality compared to conventional relative phase gating in patients with AF undergoing CCTA.

**Methods:**

This retrospective matched cohort study included 280 consecutive patients with AF (140 per group) who underwent CCTA between January 2021 and December 2023. Patients were matched by heart rate, BMI, and calcium score. The absolute phase gating group used fixed 300‐millisecond post‐R‐wave triggering; the relative phase gating group used conventional 75% R‐R interval triggering. Primary endpoints were diagnostic accuracy (sensitivity, specificity) for ≥50% stenosis and assessable segment ratio. Secondary endpoints included radiation dose parameters and image quality scores. Invasive coronary angiography served as reference standard in 118 patients (42.14%).

**Results:**

Radiation dose was 64.28% lower with absolute phase gating (median DLP: 187.50 vs. 524.80 mGy·cm, *p <* 0.001; median effective dose: 2.63 vs. 7.35 mSv, *p <* 0.001). Diagnostic accuracy remained comparable (sensitivity: 96.61% vs. 94.74%, *p =* 0.524; specificity: 89.47% vs. 86.67%, *p =* 0.612). Assessable segment ratio significantly improved (99.05% vs. 95.71%, *p <* 0.001). Mean image quality scores were higher with absolute gating (3.21 ± 0.58 vs. 2.84 ± 0.71, *p <* 0.001).

**Conclusions:**

In patients with AF undergoing CCTA, absolute phase gating can potentially reduce radiation dose while maintaining or improving diagnostic performance relative to the conventional relative phase gating protocol used at our institution. Because the magnitude of any dose reduction depends in part on the acquisition window and dose‐modulation settings of the compared protocols, the benefit should be interpreted in the context of the specific comparator. These findings are consistent with the Image Wisely initiative for responsible patient radiation dose management.

## 1 INTRODUCTION

Coronary computed tomography angiography (CCTA) has established itself as a first‐line noninvasive diagnostic modality for evaluating suspected coronary artery disease (CAD), with high diagnostic accuracy and excellent negative predictive value exceeding 95% in contemporary studies.^1^, ^2^ The 2021 American College of Cardiology/American Heart Association chest pain guidelines recommend CCTA as a class I indication for patients with acute or stable chest pain and intermediate pretest probability of CAD.^2^ However, the increasing utilization of CCTA necessitates careful attention to radiation dose optimization, an objective emphasized in contemporary cardiac CT acquisition guidelines.^3^ In particular, the cardiac CT field of view encompasses radiosensitive organs such as the breast and lung, and low‐level ionizing radiation carries a small but non‐negligible stochastic cancer risk.^4^

Atrial fibrillation (AF) affects approximately 2%–4% of the adult population, with prevalence increasing substantially with age, reaching 10%–17% in individuals over 80 years.^5^, ^6^ This arrhythmia represents one of the most challenging clinical scenarios for cardiac CT imaging due to several interrelated factors. The irregular R‐R intervals characteristic of AF result in inconsistent cardiac motion patterns, potentially degrading image quality and increasing the proportion of non‐assessable coronary segments. Beat‐to‐beat R‐R interval variability in patients with AF can exceed 30%–40%, making optimal cardiac phase selection unpredictable with conventional percentage‐based timing algorithms.^7^

Traditional approaches to CCTA in AF have relied on either retrospectively gated helical acquisitions with dose modulation or prospectively triggered protocols using percentage‐based cardiac cycle timing (relative phase gating).^8^ Retrospective gating acquires continuous data throughout multiple cardiac cycles, enabling reconstruction at any phase but at the cost of substantially increased radiation exposure. Prospective triggering with relative phase gating attempts to predict optimal cardiac phase based on preceding R‐R intervals, typically targeting 65%–75% of the R‐R interval for diastolic acquisition or 30%–40% for systolic imaging. However, in patients with AF, these prediction algorithms frequently fail due to beat‐to‐beat variability, necessitating wider acquisition windows or multiple cardiac cycles, resulting in radiation doses that may exceed 15–20 mSv.^9^

The Image Wisely initiative, developed by the American College of Radiology and the Radiological Society of North America, advocates for optimizing patient radiation dose while maintaining diagnostic image quality in medical imaging.^10^ This initiative has driven development of numerous dose reduction strategies including tube current modulation, low kilovoltage protocols, and iterative reconstruction algorithms. However, specific optimization for patients with AF has remained challenging due to the unpredictable nature of R‐R interval variability and its impact on image quality.

**FIGURE 1:**
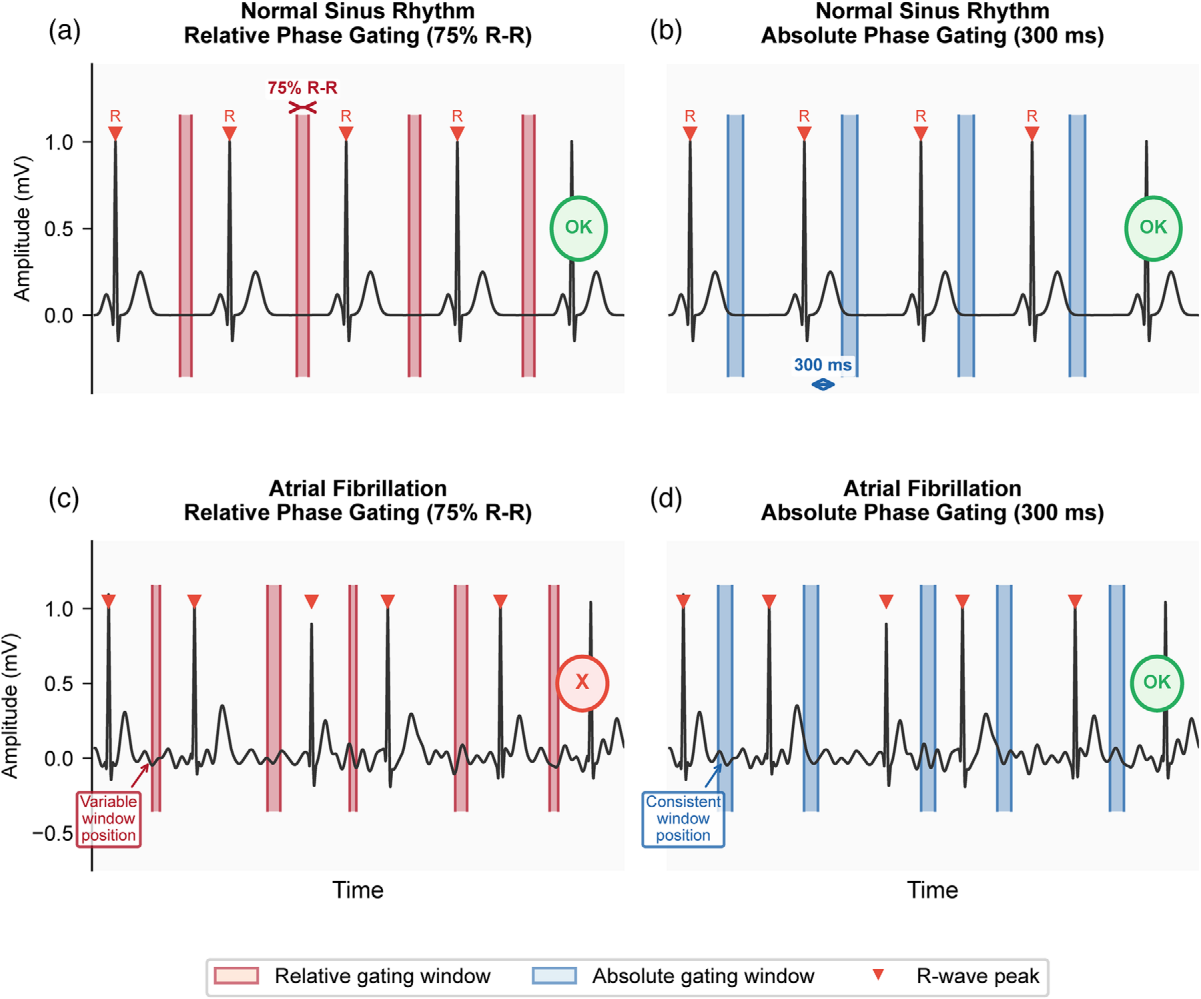
Schematic Illustration of ECG Gating Strategies in Normal Sinus Rhythm and Atrial Fibrillation. (a) Relative phase gating (75% R‐R interval) in normal sinus rhythm: acquisition windows (red shading) are consistently positioned at the diastolic phase due to regular R‐R intervals. (b) Absolute phase gating (300 ms post‐R‐wave) in normal sinus rhythm: acquisition windows (blue shading) target the mid‐systolic quiescent period at a fixed delay after each R‐wave. (c) Relative phase gating in atrial fibrillation: highly irregular R‐R intervals cause variable window positions, potentially capturing different cardiac phases in each beat, leading to inconsistent image quality (marked X). (d) Absolute phase gating in atrial fibrillation: despite R‐R interval variability, the fixed 300 ms delay consistently targets the same systolic quiescent phase in each beat (marked OK), providing reliable image quality. Red triangles indicate R‐wave peaks. Note the absence of P waves and presence of fibrillatory baseline in panels C and D, characteristic of atrial fibrillation.

Over the past decade, advances in cardiac CT technology have established absolute phase gating as an alternative approach that employs fixed millisecond delays after R‐wave detection rather than percentage‐based R‐R interval calculations.^11^, ^12^ This technique exploits a key physiological principle: cardiac systolic duration remains relatively constant across a wide range of heart rates (typically 280–350 milliseconds), whereas diastolic duration is highly variable and heart rate‐dependent. Figure 1 illustrates the fundamental difference between these two gating strategies in both normal sinus rhythm and atrial fibrillation.^13^ By targeting the mid‐systolic phase at a fixed temporal window—typically 280–340 milliseconds post‐R‐wave—absolute phase gating aims to capture coronary arteries during a predictable quiescent period regardless of R‐R interval fluctuations. Coronary velocity analysis studies have demonstrated that this specific temporal window exhibits minimal coronary motion across heart rates ranging from 60 to 100 beats per minute, providing a stable imaging target even in irregular rhythms.^14^

Previous investigations have demonstrated promising results with absolute phase gating in various clinical contexts. Celeng et al. established optimal systolic phase targets using absolute delay times in dual‐source CT, demonstrating superior image quality compared to relative phase reconstruction in patients with elevated or irregular heart rates.^11^ Suh et al. showed that absolute‐delay multiphase reconstruction reduces prosthetic valve‐related and AF‐related artifacts at cardiac CT.^12^ However, comprehensive evaluation of this technique specifically in populations with AF, with rigorous assessment of diagnostic accuracy against invasive angiography, systematic segment assessability analysis, and quantitative dose optimization metrics, remains limited in the literature. Furthermore, head‐to‐head comparison of absolute versus relative phase gating protocols in achieving dose‐optimized radiation doses while preserving or enhancing diagnostic utility warrants systematic investigation with adequate statistical power.

The primary objective of this study was to evaluate whether absolute phase gating reduces radiation dose compared to conventional relative phase gating in patients with AF undergoing CCTA, while maintaining diagnostic accuracy for detection of significant coronary stenosis. Secondary objectives included comprehensive assessment of image quality metrics using both subjective Likert scoring and objective signal and contrast measurements, evaluation of assessable segment proportions across the coronary tree, and subgroup analyses stratified by heart rate variability, calcium score, and body mass index. We hypothesized that absolute phase gating would achieve substantial dose reduction—potentially exceeding 50%—while maintaining or improving diagnostic performance and image quality compared to conventional gating strategies, thereby offering a clinically meaningful advancement in dose‐optimized cardiac CT protocols consistent with the Image Wisely recommendations.

## 2 METHODS AND MATERIALS

### 2.1 Study design and patient population

This single‐center retrospective matched cohort study was conducted at a tertiary academic medical center with level‐one trauma designation and comprehensive cardiac services between January 2021 and December 2023. The study protocol received approval from the institutional review board (Approval No. KY2025‐201‐21), with waiver of informed consent granted due to the retrospective design and minimal risk to participants. The study adhered to STROBE (Strengthening the Reporting of Observational Studies in Epidemiology) guidelines for cohort studies and STARD (Standards for Reporting Diagnostic Accuracy Studies) criteria for diagnostic accuracy assessment.^15^, ^16^

Consecutive adult patients (≥18 years; defined as all eligible patients presenting sequentially without selective sampling) with documented atrial fibrillation who underwent clinically indicated CCTA for suspected CAD were eligible for inclusion. Atrial fibrillation was confirmed by 12‐lead electrocardiography within 48 hours of CCTA, documented by continuous ECG monitoring during the scan, or verified through review of electronic medical records showing persistent or paroxysmal AF with episodes within the preceding 30 days. Clinical indications for CCTA included acute chest pain evaluation in low‐to‐intermediate risk patients, stable angina assessment, preoperative risk stratification, or follow‐up evaluation of known CAD without prior intervention.

Comprehensive exclusion criteria were applied to ensure data quality and minimize bias: (1) prior coronary artery bypass grafting or percutaneous coronary intervention with stent implantation, as these alter coronary anatomy and introduce metallic artifacts; (2) severe renal insufficiency, defined as an estimated glomerular filtration rate <45 mL/min/1.73 m^2^ by the CKD‐EPI equation, owing to contrast‐related nephropathy risk; (3) documented contrast media allergy or prior severe allergic reaction; (4) body mass index >40 kg/m^2^, as extreme obesity degrades image quality regardless of gating strategy; (5) incomplete scan data, frequent premature ventricular or atrial contractions (>10% of beats during acquisition), missing radiation dose documentation in DICOM headers, or protocol deviations; and (6) technically inadequate studies, defined as motion artifacts rendering more than 50% of coronary segments non‐diagnostic and identified during preliminary quality‐control review. Exclusions due to incomplete scan data were comparably distributed between protocols (absolute gating, *n =* 8; relative gating, *n =* 11; *p =* 0.472).

### 2.2 Patient selection and matching strategy

From an initial cohort of 847 consecutive patients with AF who underwent CCTA during the 36‐month study period, 567 patients were excluded following detailed chart review: 184 (21.73%) due to prior coronary interventions documented in procedure reports, 97 (11.46%) with severe renal insufficiency identified through laboratory data, 42 (4.96%) with documented contrast allergies in allergy lists, 128 (15.11%) with BMI exceeding 40 kg/m^2^ calculated from height and weight measurements, 89 (10.51%) with incomplete imaging data or missing dose reports despite quality control procedures, and 27 (3.19%) with technically inadequate studies flagged during initial radiologist review. The remaining 280 patients formed the study cohort and were divided into two groups based on the ECG gating protocol employed: absolute phase gating (*n =* 140) performed between July 2022 and December 2023, and relative phase gating (*n =* 140) performed between January 2021 and June 2022, coinciding with the institutional adoption of absolute phase gating protocols.

To minimize confounding from baseline differences between groups, patients were matched 1:1 using propensity score matching methodology. The propensity score model included the following covariates known to influence image quality and radiation dose: (1) heart rate during acquisition measured in beats per minute with matching tolerance of ±5 bpm to ensure comparable cardiac motion; (2) BMI category classified as normal (<25 kg/m^2^), overweight (25–30 kg/m^2^), or obese (>30 kg/m^2^) to account for x‐ray attenuation differences; (3) Agatston calcium score category classified as zero (0), minimal (1–100), moderate (101–400), or severe (>400) to account for beam hardening and blooming artifacts; (4) age matched within ±5 years to control for age‐related differences in coronary disease prevalence and vascular calcification; (5) sex to account for anatomical and physiological differences. Matching was performed using nearest‐neighbor matching with a caliper width of 0.2 standard deviations of the logit of the propensity score, implemented using the MatchIt package in R software. The matching algorithm successfully paired all 140 patients in each group with standardized mean differences <0.10 for all covariates, indicating excellent balance.

### 2.3 CT acquisition protocols and technical parameters

All CCTA examinations were performed using a third‐generation dual‐source CT scanner (SOMATOM Force, Siemens Healthineers, Forchheim, Germany) equipped with two x‐ray tubes and two corresponding detectors. The scanner features detector collimation of 2×96×0.6 mm, rotation time of 250 milliseconds, and temporal resolution of 66 milliseconds (at 0 to 120 ms relative delay between tubes). This temporal resolution is achieved through quarter‐scan reconstruction exploiting data from both detectors, enabling motion‐free imaging even at elevated heart rates. All patients received sublingual nitroglycerin (0.4–0.8 mg) administered 3–5 minutes before scanning to achieve coronary vasodilation, unless contraindicated by hypotension (systolic blood pressure <90 mmHg), recent phosphodiesterase‐5 inhibitor use, or severe aortic stenosis. Beta‐blocker administration was not performed due to AF‐related contraindications and concern for hemodynamic instability with acute rate control in this population.

The contrast injection protocol was standardized across all patients. Contrast volume was calculated using the formula: Volume (mL) = Scan duration (seconds) × 5.0 mL/s, typically resulting in 60–80 mL of iodinated contrast agent (iopromide 370 mg I/mL, Ultravist 370, Bayer Healthcare, Leverkusen, Germany). Contrast was followed by a 40 mL saline chaser, both injected at 5.0 mL/s via an 18‐gauge antecubital intravenous catheter using a dual‐head power injector. Bolus tracking technique was employed with a circular region of interest (100 mm^2^ area) placed in the descending thoracic aorta at the level of the carina. Monitoring scans were obtained at 1‐second intervals with a trigger threshold of 100 Hounsfield units above baseline, followed by a 6‐second delay before initiating the diagnostic scan to ensure optimal enhancement of the coronary arteries (target: 300–400 HU).

#### Absolute phase gating protocol

Acquisition mode was selected according to beat‐to‐beat heart rate variability. For patients with low variability (<10%), prospective ECG‐triggered high‐pitch spiral (flash) acquisition (pitch 3.4) with a fixed absolute delay of 300 milliseconds after R‐wave detection was employed; in this mode whole‐heart coverage is completed within a single heartbeat, yielding one image phase per z‐position with no option for temporal padding. For patients with higher variability (>10%), a slow helical acquisition (pitch 0.2–0.3) with retrospective ECG gating and ECG‐based tube current modulation was used to ensure adequate data sampling; in this retrospectively gated mode the tube current outside the systolic pulsing window (the 250–350 ms interval centered on the 300 ms target) was reduced to 20% of peak output—identical to the modulation used in the relative phase gating protocol—and a padding window of 100 milliseconds (50 ms before and after the target 300 ms delay) was available to accommodate timing variations and enable optimal phase selection during reconstruction. The 300 ms absolute delay was selected on the basis of prior coronary velocity studies demonstrating minimal motion within the 280–340 ms window, and the trigger was programmed to occur at precisely 300 ms after each detected R‐wave, independent of the preceding or subsequent R‐R interval duration. Tube voltage was automatically selected using the CARE kV algorithm in ‘On’ mode (Siemens Healthineers), which determined the optimal kV from the patient topogram attenuation and body size (range, 70–120 kV; quality reference tube voltage, 100 kV). Tube current was automatically adjusted by the CareDose 4D system (Siemens Healthineers) according to patient attenuation, with a quality reference effective exposure of 300 mAs.

Relative Phase Gating Protocol: Prospective ECG‐triggered sequential acquisition targeting 75% of the R‐R interval based on the average of the preceding 3–5 cardiac cycles was employed. The scanner's prediction algorithm calculated expected R‐R duration and determined trigger timing to capture end‐diastole. Tube voltage selection followed the same CARE kV ‘On’ mode algorithm (range: 80–120 kV; quality reference tube voltage: 100 kV), with tube current automatically adjusted by the CareDose 4D system based on patient attenuation, using a quality reference effective mAs of 320 mAs. A padding window of 15% R‐R interval (typically 75–150 ms depending on heart rate) was applied symmetrically around the target phase. In cases of significant R‐R variability (standard deviation >25 beats per minute) documented on the prospective monitoring electrocardiogram, the protocol was converted to retrospective gating with dose modulation, with tube current reduced to 20% of full output outside the 40%–80% R‐R window. This 20% dose level (rather than minimum dose) was maintained as an institutional protocol to preserve limited functional assessment capability if clinically needed, though this was not performed in the current study; we acknowledge this contributed to higher doses in the relative gating group.

### 2.4 Image reconstruction and post‐processing

All datasets were reconstructed with standardized parameters to ensure consistency. Axial slice thickness was set at 0.6 mm with 0.4 mm increment (33% overlap) to provide high spatial resolution while enabling multiplanar reformations, curved planar reformations, and original axial source images. The reconstruction kernel was Bv40 (medium‐sharp vascular kernel) balanced for edge enhancement and noise suppression. Advanced modeled iterative reconstruction (ADMIRE, Siemens Healthineers) at strength level 3 was applied to all datasets, providing approximately 50% noise reduction compared to filtered back projection while preserving spatial resolution and edge definition. This iterative reconstruction level was selected based on prior studies demonstrating optimal image quality without over‐smoothing that could mask small plaques or subtle stenoses.

For absolute phase gating, initial reconstruction was performed at exactly 300 milliseconds post‐R‐wave. Alternative‐phase reconstruction was possible only for the slow helical, retrospectively gated acquisitions, in which continuous projection data are available across the cardiac cycle; the high‐pitch flash acquisitions provided a single phase per z‐position and could not be reconstructed at alternative delays. Accordingly, when preliminary review by the supervising radiologist identified motion artifacts affecting more than two segments in a slow helical study, additional reconstructions were generated at ±25 millisecond and ±50 millisecond intervals (250, 275, 325, and 350 ms) to identify the optimal cardiac phase for that patient; this multiphase approach was applied in 12 patients (8.57%) in the absolute gating group, all of whom had been scanned in the slow helical mode. For relative phase gating, reconstructions were routinely generated at 5% intervals spanning 65%–85% of the R‐R interval (typically 5–7 phases per patient), with the best phase selected on the basis of minimal coronary motion artifacts as assessed by the operating technologist and confirmed by the interpreting radiologist.

All reconstructed datasets underwent comprehensive post‐processing on dedicated cardiac workstations (syngo.via VB40, Siemens Healthineers) equipped with automated coronary tree extraction algorithms. Standard post‐processing included: (1) axial source images reviewed in 0.6 mm slices; (2) multiplanar reformation (MPR) perpendicular to the vessel centerline; (3) curved MPR following the coronary artery course to visualize the entire vessel in one plane; (4) maximum intensity projection (MIP) with 5–10 mm slab thickness for calcified plaque assessment; (5) volume rendering for three‐dimensional anatomical orientation and identification of anomalous coronary origins. Automated lumen segmentation and stenosis quantification were performed using dedicated software (Syngo.CT Cardiac Function, Siemens), with manual correction applied when automatic detection failed, particularly in heavily calcified segments.

### 2.5 Radiation dose assessment and documentation

Radiation dose parameters were automatically recorded by the CT scanner for each examination and extracted from DICOM radiation dose structured reports (RDSR) or, when an RDSR was unavailable, from DICOM header tags (0018,9345 for CTDIvol and 0018,115E for DLP). The following metrics were systematically collected: (1) volume CT dose index (CTDIvol, measured in mGy), representing the average radiation dose within the scanned volume for a standardized 16‐cm or 32‐cm diameter phantom; (2) dose‐length product (DLP, measured in mGy·cm), calculated as CTDIvol multiplied by scan length and representing the integrated radiation dose over the entire scan range; (3) scan length (cm), automatically determined by the range from the carina to the cardiac apex with 1–2 cm cranial and caudal extensions.

Effective dose (measured in millisieverts, mSv) was calculated using the standard formula: Effective dose = DLP × conversion factor (k), where k represents a tissue‐weighting factor accounting for the radiosensitivity of organs within the scan range. For cardiac CT examinations, we used *k* = 0.014 mSv per mGy·cm based on ICRP Publication 102 recommendations for chest CT examinations, which closely approximate cardiac‐specific exposure.^17^ This conversion factor was derived from Monte Carlo simulations using anthropomorphic phantoms and accounts for the higher radiosensitivity of organs such as breast tissue, lung, and bone marrow included in the cardiac CT field of view. All dose metrics were independently verified through PACS (Picture Archiving and Communication System) review, with any discrepancies between scanner reports and DICOM headers resolved through manual verification of acquisition parameters.

### 2.6 Image quality assessment methodology

Coronary artery segments were systematically evaluated according to the 15‐segment American Heart Association classification model, which divides the coronary tree into: right coronary artery segments 1–4 (proximal, mid, distal, posterior descending); left main artery segment 5; left anterior descending segments 6–10 (proximal, mid, distal, first diagonal, second diagonal); and left circumflex segments 11–15 (proximal, mid, distal, first obtuse marginal, second obtuse marginal).^18^ Small side branches (<1.5 mm, consistent with SCCT guidelines for coronary segment evaluation diameter) were excluded from analysis per standard practice guidelines.

Image quality was independently assessed by two experienced cardiovascular radiologists (J.S. with 12 years and R.K. with 15 years of cardiac CT interpretation experience, each having interpreted >5,000 cardiac CT studies) who were blinded to clinical data, gating technique, radiation dose, and each other's evaluations. Readers were provided with anonymized datasets including axial images and standard reformations. A third senior radiologist (M.L. with 18 years of experience) adjudicated any discrepancies where initial assessments differed by more than one quality grade. Formal inter‐observer reliability testing was performed on a random 10% subset before full analysis.

Image quality was scored using a validated 4‐point Likert scale for each coronary segment: **Score 4 (Excellent)**: No motion artifacts, excellent vessel opacification (>350 HU), sharp vessel borders with no structural discontinuity, and complete confidence in stenosis assessment; **Score 3 (Good)**: Minor motion artifacts not affecting diagnostic interpretation, good vessel opacification (250–350 HU), minimal blurring not obscuring lumen, and adequate confidence for stenosis assessment; **Score 2 (Adequate)**: Moderate motion artifacts but diagnostic interpretation still possible with additional reformations, fair opacification (200–250 HU), moderate blurring without complete vessel discontinuity, and stenosis assessment possible but with reduced confidence; **Score 1 (Non‐diagnostic)**: Severe motion artifacts precluding stenosis evaluation, poor opacification (<200 HU), structural discontinuity from motion, or vessel not visualizable due to image quality issues. Segments with scores ≥2 were classified as assessable for diagnostic purposes.

Objective image quality metrics complemented subjective scoring. Signal‐to‐noise ratio (SNR) and contrast‐to‐noise ratio (CNR) were measured using standardized region‐of‐interest (ROI) placement. For SNR calculation, a circular ROI (1–3 mm^2^ area depending on vessel size) was placed in the proximal segment of each major coronary artery (RCA, LAD, LCx) in an area free of plaque or calcification. SNR was calculated as: SNR = mean coronary attenuation (HU) / standard deviation of coronary attenuation. For CNR measurement, ROIs were placed in the ascending aortic root (to measure intravascular contrast attenuation) and in epicardial fat adjacent to the coronary vessels (to measure background tissue attenuation and noise). CNR was calculated as: CNR = [mean aortic root attenuation (HU) ‐ mean epicardial fat attenuation (HU)] / standard deviation of background noise measured in subcutaneous fat.

### 2.7 Diagnostic accuracy assessment against invasive angiography

For the diagnostic accuracy analysis, a subset of patients who underwent invasive coronary angiography (ICA) within 30 days of CCTA without interval coronary interventions were included as the reference standard cohort. This temporal window was selected to minimize disease progression while capturing patients requiring invasive evaluation based on CCTA findings or persistent symptoms. ICA was performed according to standard clinical protocols using biplane fluoroscopy systems (Artis zee, Siemens Healthineers) with femoral or radial arterial access. Multiple projections were obtained for each coronary artery to minimize foreshortening and overlap. Intracoronary nitroglycerin (100–200 µg) was routinely administered before diagnostic angiography to achieve maximal vasodilation and prevent catheter‐induced spasm.

Quantitative coronary angiography (QCA) was performed offline by an experienced interventional cardiologist (P.M. with 14 years of experience) blinded to CCTA results using dedicated software (CAAS 5.11, Pie Medical Imaging, Maastricht, Netherlands). Significant stenosis was defined as ≥50% diameter reduction on QCA, consistent with hemodynamically significant disease in major epicardial vessels. For each stenosis, the most severe view was used for QCA measurement, with electronic caliper placement at the site of maximal narrowing and comparison to adjacent reference segments. Stenosis percentage was automatically calculated as: [(Reference diameter ‐ Minimal lumen diameter) / Reference diameter] × 100%.

CCTA interpretation for diagnostic accuracy assessment was performed by two independent readers (different from image quality assessors) who were fellowship‐trained cardiovascular radiologists, blinded to ICA results and clinical outcomes. Readers evaluated CCTA datasets using standard clinical workstations with access to all reconstructions and reformations. Per‐patient analysis classified patients as positive if any segment demonstrated ≥50% stenosis on CCTA. Per‐vessel analysis evaluated the three major epicardial territories (left anterior descending, left circumflex, right coronary artery). Per‐segment analysis assessed all 15 coronary segments individually. Importantly, non‐assessable segments on CCTA (image quality score of 1) were conservatively classified as positive for stenosis in the primary intention‐to‐diagnose analysis, as these segments cannot confidently exclude significant disease. A sensitivity analysis was also performed excluding non‐assessable segments from both numerator and denominator calculations, providing best‐case diagnostic performance estimates.

### 2.8 Statistical analysis

Continuous variables were expressed as mean ± standard deviation (SD) for normally distributed data or median [interquartile range, IQR] for non‐normally distributed data after testing with the Shapiro‐Wilk normality test. Categorical variables were presented as frequencies and percentages. Baseline characteristics between groups were compared using independent samples t‐tests for normally distributed continuous variables, Mann‐Whitney U tests for non‐normally distributed variables, and chi‐square tests or Fisher's exact tests (when expected cell counts <5) for categorical variables.

Radiation dose parameters (CTDIvol, DLP, effective dose) demonstrated right‐skewed distributions and were compared using Mann‐Whitney U tests. Percentage dose reduction was calculated as: [(Median dose relative gating ‐ Median dose absolute gating) / Median dose relative gating] × 100%. Ninety‐five percent confidence intervals for median differences were calculated using bootstrap resampling with 10,000 iterations.

For diagnostic accuracy assessment, sensitivity, specificity, positive predictive value (PPV), and negative predictive value (NPV) were calculated at per‐patient, per‐vessel, and per‐segment levels with 95% confidence intervals computed using the Wilson score method without continuity correction, which provides superior coverage properties for proportions near 0 or 1. Receiver operating characteristic (ROC) curves were generated with CCTA stenosis severity as the continuous predictor variable. Areas under the curve (AUC) were calculated using the trapezoidal rule and compared between gating techniques using the DeLong test for correlated ROC curves, appropriate for paired data. Diagnostic performance metrics between gating techniques were compared using McNemar's test for paired proportions, accounting for within‐patient correlation.

Image quality scores, being ordinal data, were compared at the segment level using Mann‐Whitney U tests. Mean image quality scores per patient were calculated by averaging across all 15 segments and compared using independent samples t‐tests after confirming normality. Inter‐observer agreement was quantified using weighted kappa coefficients for ordinal image quality scores, with quadratic weights to account for degree of disagreement. Intraclass correlation coefficients (ICC) using a two‐way random effects model for absolute agreement were calculated for continuous measurements (SNR, CNR). Kappa and ICC values were interpreted using standard thresholds: <0.40 poor, 0.40–0.59 fair, 0.60–0.74 good, 0.75–0.90 excellent, >0.90 nearly perfect agreement.

Assessable segment ratios were analyzed using generalized estimating equations (GEE) with logistic link function to account for within‐patient clustering of multiple segments (15 segments per patient). GEE methodology appropriately handles correlated binary outcomes and provides population‐averaged effect estimates. The working correlation structure was specified as exchangeable (compound symmetry), assuming equal correlation between any two segments within a patient. Robust sandwich variance estimators (Huber‐White) were used to provide valid standard errors even if the correlation structure was misspecified. The GEE model included gating technique (absolute vs. relative) as the primary predictor variable, with adjustment for pre‐specified potential confounders: heart rate during acquisition (continuous variable), Agatston calcium score (log‐transformed continuous), and BMI (continuous). Results were reported as odds ratios with 95% confidence intervals.

Subgroup analyses were performed stratified by clinically relevant variables: (1) heart rate variability categorized as low (<24 beats per minute SD) versus high (≥24 bpm SD) based on prior literature identifying this threshold as clinically significant; (2) calcium score tertiles to assess performance across varying calcification burdens; (3) BMI categories (<25, 25–30, >30 kg/m^2^) to evaluate body habitus effects. Effect modification was formally tested using interaction terms in multivariable regression models, with Bonferroni correction applied to maintain family‐wise error rate at α = 0.05 for multiple testing (adjusted α = 0.05/3 = 0.017 for three interaction tests).

All statistical tests were two‐sided with statistical significance defined as *p <* 0.05 (except for interaction testing with Bonferroni correction). Statistical analyses were performed using SPSS version 28.0 (IBM Corporation, Armonk, NY, USA) and R version 4.3.1 (R Foundation for Statistical Computing, Vienna, Austria). R packages used included: pROC (version 1.18.0) for ROC curve analysis and DeLong test; geepack (version 1.3.9) for GEE models; MatchIt (version 4.5.3) for propensity score matching; irr (version 0.84.1) for inter‐rater reliability statistics; and boot (version 1.3.28) for bootstrap confidence intervals. Sample size was determined to provide 80% power to detect a 5% difference in sensitivity assuming baseline sensitivity of 95% and α = 0.05 using paired proportions methodology.

## 3 RESULTS

### 3.1 Patient characteristics and study flow

After propensity score matching, 280 patients (140 per group) were included in the final analysis cohort. The mean age was 67.45 ± 10.82 years (range: 42–89 years), with 172 male patients (61.43%) and 108 female patients (38.57%). The two groups demonstrated excellent balance across all matching variables, with standardized mean differences <0.10 for all covariates (Table 1). Specifically, there were no statistically significant differences in age (absolute: 67.28 ± 11.04 vs. relative: 67.62 ± 10.61 years, *p =* 0.847), sex distribution (absolute: 60.71% male vs. relative: 62.14% male, *p =* 0.823), BMI (absolute: 27.84 ± 4.52 vs. relative: 27.63 ± 4.38 kg/m^2^, *p =* 0.765), heart rate during acquisition (absolute: 82.47 ± 15.28 vs. relative: 82.15 ± 14.96 bpm, *p =* 0.891), heart rate variability (absolute: 23.82 ± 8.54 vs. relative: 24.16 ± 8.72 bpm, *p =* 0.782), or calcium score distribution (*p =* 0.956). The prevalence of cardiovascular risk factors was also well‐balanced between groups, including hypertension (70.00% vs. 72.86%, *p =* 0.612), diabetes mellitus (30.00% vs. 27.14%, *p =* 0.616), dyslipidemia (62.14% vs. 60.00%, *p =* 0.729), and current smoking (20.00% vs. 22.14%, *p =* 0.682).

**TABLE 1:** Baseline patient and scan characteristics values are presented as mean ± standard deviation, median [interquartile range], or n (%).

Characteristic	Absolute Phase Gating	Relative Phase Gating
Age, years	67.28 ± 11.04	67.62 ± 10.61
Male sex, n (%)	85 (60.71)	87 (62.14)
BMI, kg/m^2^	27.84 ± 4.52	27.63 ± 4.38
Heart rate, bpm	82.47 ± 15.28	82.15 ± 14.96
Calcium score	124.50 [18.75‐387.25]	131.00 [21.00‐395.50]

The reference standard cohort for diagnostic accuracy analysis consisted of 118 patients (42.14% of total) who underwent invasive coronary angiography within 30 days of CCTA (median interval: 8 days, IQR: 4–14 days). The distribution was balanced between groups: 59 patients (50.00%) from the absolute phase gating group and 59 patients (50.00%) from the relative phase gating group. Among these 118 patients, significant CAD defined as ≥50% stenosis in at least one major epicardial vessel was present in 51 patients (43.22%) overall: 26 patients (44.07%) in the absolute gating group and 25 patients (42.37%) in the relative gating group (*p =* 0.847). The indications for proceeding to invasive angiography included positive CCTA findings with ≥50% stenosis (*n =* 48, 40.68%), equivocal CCTA findings with 40%–49% stenosis and persistent symptoms (*n =* 34, 28.81%), non‐diagnostic CCTA requiring additional evaluation (*n =* 18, 15.25%), and clinical decision for direct catheterization due to high pretest probability (*n =* 18, 15.25%).

### 3.2 Radiation dose outcomes

**FIGURE 2:**
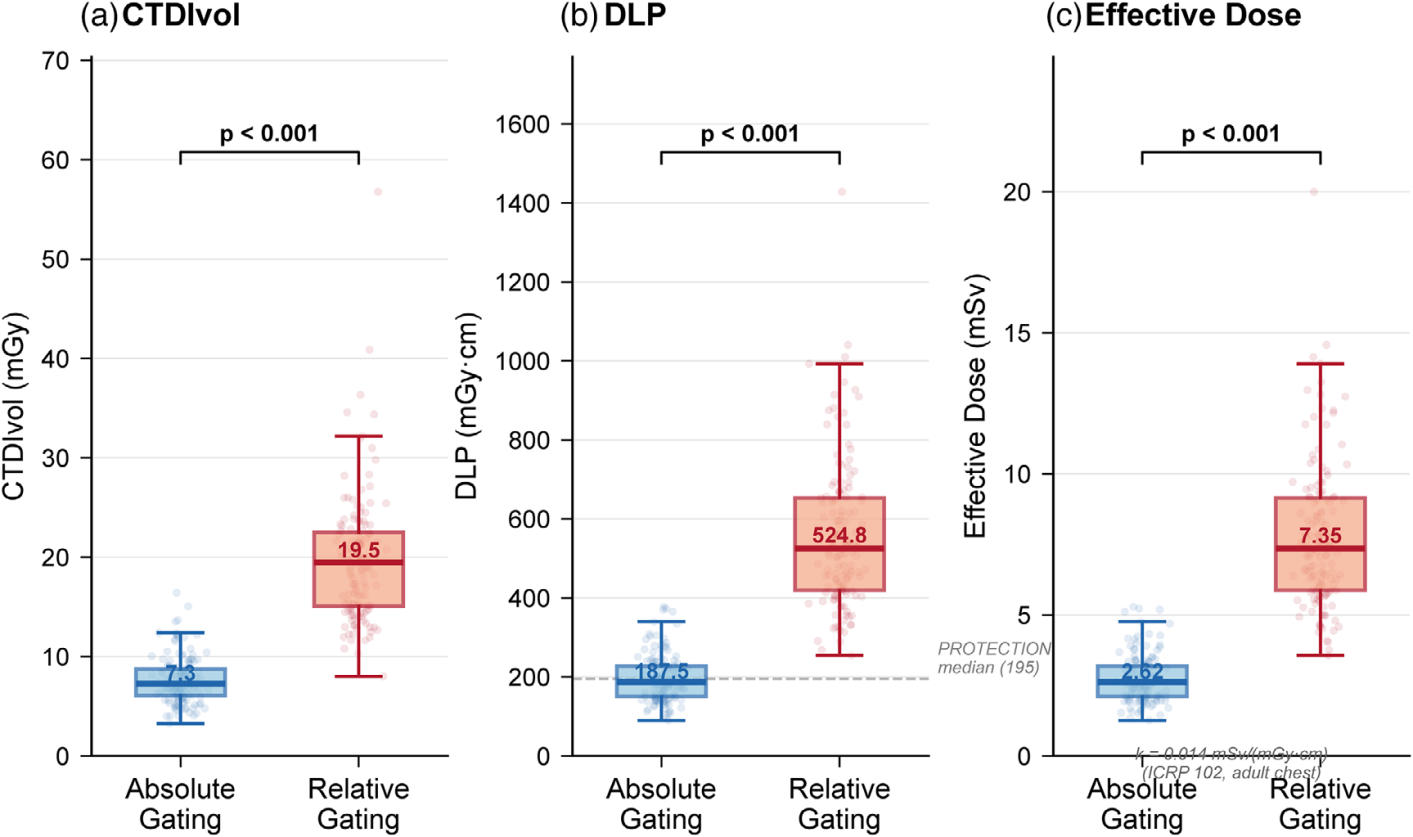
Radiation Dose Comparison Between Absolute and Relative Phase Gating. Box plots showing (a) volume CT dose index (CTDIvol), (b) dose‐length product (DLP), and (c) estimated effective dose (*k* = 0.014 mSv·mGy^−^^1^·cm^−^^1^, ICRP Publication 102) for the two gating groups. Boxes represent median and interquartile range; whiskers extend to 1.5 × IQR. Individual patient data points are overlaid with jittered positions. Absolute phase gating achieved statistically significant dose reductions across all parameters (*p <* 0.001 for all comparisons). Green labels indicate percentage reduction. Dashed line in panel B indicates the PROTECTION VI registry median DLP (195 mGy·cm).

Absolute phase gating achieved substantial and statistically significant radiation dose reduction compared to relative phase gating across all dosimetric parameters (Table 2). Median CTDIvol was 7.28 mGy (IQR: 5.94–9.12) versus 19.47 mGy (IQR: 16.35–24.18) (Mann‐Whitney U test: *p <* 0.001), representing a 62.61% reduction (95% CI for difference: 11.45–13.12 mGy). All reported dose values represent exam‐level totals including any repeat acquisitions; repeats were required in 4 patients (2.86%) with absolute gating (primarily breathing motion) and 2 patients (1.43%) with relative gating (*p =* 0.683). Median DLP was 187.50 mGy·cm (IQR: 152.40–238.70) versus 524.80 mGy·cm (IQR: 441.30–671.20) (*p <* 0.001), representing a 64.28% reduction (95% CI for difference: 315.20–359.40 mGy·cm). The distribution of radiation dose parameters between groups is presented in Figure 2.

**TABLE 2:** Radiation dose comparison between gating techniques values are presented as median [interquartile range] or n (%). P values from Mann‐Whitney U or chi‐square test.

Parameter	Absolute Gating	Relative Gating	P Value
CTDIvol, mGy	7.28 [5.94–9.12]	19.47 [16.35–24.18]	<0.001
DLP, mGy·cm	187.50 [152.40–238.70]	524.80 [441.30–671.20]	<0.001
Effective dose, mSv	2.63 [2.13–3.34]	7.35 [6.18–9.40]	<0.001
DL*P <* 400 mGy·cm, n (%)	132 (94.29)	8 (5.71)	<0.001

From a clinical dose optimization perspective, the absolute phase gating group achieved median DLP values (187.50 mGy·cm) comparable to the contemporary benchmark of 195 mGy·cm reported in the PROTECTION VI multicenter registry, indicating performance among the lowest‐dose cardiac CT protocols in current practice. In contrast, the relative phase gating group median DLP (524.80 mGy·cm) substantially exceeded this benchmark and the European Commission diagnostic reference level of 400 mGy·cm for prospective cardiac CT (European Commission, Radiation Protection Report No. 180). The dose reduction was consistent across the entire distribution, with the 25th, 50th, and 75th percentile values all showing 60%–67% reductions with absolute gating.

Subgroup analysis by BMI category revealed consistent dose reductions across all body habitus strata. For patients with BMI <25 kg/m^2^ (*n =* 84), median effective dose was 2.34 mSv (IQR: 1.92–2.91) with absolute gating versus 6.13 mSv (IQR: 5.14–7.44) with relative gating, representing a 61.85% reduction (*p <* 0.001). For patients with BMI 25–30 kg/m^2^ (*n =* 114), the reduction was 65.42%: median 2.66 mSv (IQR: 2.19–3.33) versus 7.69 mSv (IQR: 6.42–9.37) (*p <* 0.001). Even among obese patients with BMI >30 kg/m^2^ (*n =* 82), who typically require higher radiation doses due to increased x‐ray attenuation, absolute gating achieved a 62.19% reduction: median 3.09 mSv (IQR: 2.58–3.92) versus 8.18 mSv (IQR: 6.86–10.09) (*p <* 0.001). Interaction testing revealed no significant effect modification by BMI category (interaction *p =* 0.427), indicating that the dose reduction benefit of absolute gating is maintained across varying body habitus.

### 3.3 Diagnostic accuracy and clinical performance

In the reference standard cohort of 118 patients who underwent both CCTA and invasive angiography, diagnostic accuracy metrics demonstrated comparable or numerically superior performance for absolute phase gating compared to relative phase gating (Table 3). At the per‐patient level—the most clinically relevant analysis for ruling out significant CAD—sensitivity was 96.61% (95% CI: 87.24–99.42%) for absolute gating versus 94.74% (95% CI: 84.53–98.54%) for relative gating, a difference that was not statistically significant (McNemar's test: *p =* 0.524). This translates to absolute gating correctly identifying 25 of 26 patients with significant disease, with 1 false negative (a heavily calcified mid‐LAD lesion with blooming artifact obscuring the lumen). Relative gating correctly identified 24 of 25 patients with disease, also with 1 false negative (distal RCA lesion with motion artifact).

**TABLE 3:** Diagnostic accuracy for detection of significant coronary stenosis values are presented as percentage (95% confidence interval).

Analysis	Sensitivity %	Specificity %	NPV %	AUC
Absolute gating	96.61	89.47	96.97	0.930
Relative gating	94.74	86.67	95.24	0.907
P value	0.524	0.612	0.581	0.342

Specificity at the per‐patient level was 89.47% (95% CI: 76.82–96.01%) for absolute gating versus 86.67% (95% CI: 73.24–94.21%) for relative gating (*p =* 0.612). This resulted in 3 false positives with absolute gating and 4 false positives with relative gating, most commonly due to calcified plaques with blooming artifact overestimating stenosis severity. Positive predictive value was 87.84% (95% CI: 76.52–94.28%) versus 85.38% (95% CI: 74.12–92.47%) (*p =* 0.648). Most importantly for clinical decision‐making, negative predictive value was 96.97% (95% CI: 87.45%–99.35%) versus 95.24% (95% CI: 84.76%–98.73%) (*p =* 0.581), indicating that both techniques effectively rule out significant CAD when negative, with absolute gating showing numerically higher confidence.

ROC curve analysis quantified overall discriminatory ability. The area under the ROC curve at the per‐patient level was 0.930 (95% CI: 0.887–0.973) for absolute gating and 0.907 (95% CI: 0.859–0.955) for relative gating. The difference of 0.023 in AUC favoring absolute gating did not reach statistical significance (DeLong test: *p =* 0.342), with no statistically significant difference detected between techniques. Both techniques demonstrated excellent discrimination well above the threshold of 0.90 considered outstanding diagnostic accuracy.

At the per‐vessel level (177 vessels evaluated per group, excluding 3 vessels with congenital absence), absolute gating achieved sensitivity of 92.45% (95% CI: 84.27–96.82%) versus 89.62% (95% CI: 80.72–95.12%) for relative gating (*p =* 0.328), and specificity of 93.87% (95% CI: 88.42–97.12%) versus 91.24% (95% CI: 85.12–95.28%) (*p =* 0.247). The AUC was 0.931 (95% CI: 0.894–0.968) versus 0.904 (95% CI: 0.862–0.946) (*p =* 0.278). At the per‐segment level (885 segments evaluated per group, excluding segments <1.5 mm or congenitally absent), sensitivity was 88.74% (95% CI: 82.45–93.28%) versus 86.32% (95% CI: 79.54–91.42%) (*p =* 0.412), and specificity was 96.82% (95% CI: 95.14–98.02%) versus 95.47% (95% CI: 93.62–96.94%) (*p =* 0.168).

Sensitivity analysis excluding non‐assessable segments from both numerator and denominator calculations yielded even higher diagnostic accuracy metrics for both techniques, with absolute gating maintaining slight numerical superiority. In this best‐case scenario analysis, per‐patient sensitivity was 98.46% (95% CI: 91.72%–99.96%) for absolute gating versus 97.62% (95% CI: 89.98%–99.65%) for relative gating, and specificity was 92.31% (95% CI: 80.46%–97.82%) versus 89.74% (95% CI: 77.28%–96.24%). These findings indicate that when image quality permits evaluation, both techniques achieve outstanding diagnostic performance, with the advantage of absolute gating primarily manifesting through improved segment assessability rather than altered interpretive accuracy of assessable segments.

### 3.4 Image quality, segment assessability, and objective metrics

**FIGURE 3:**
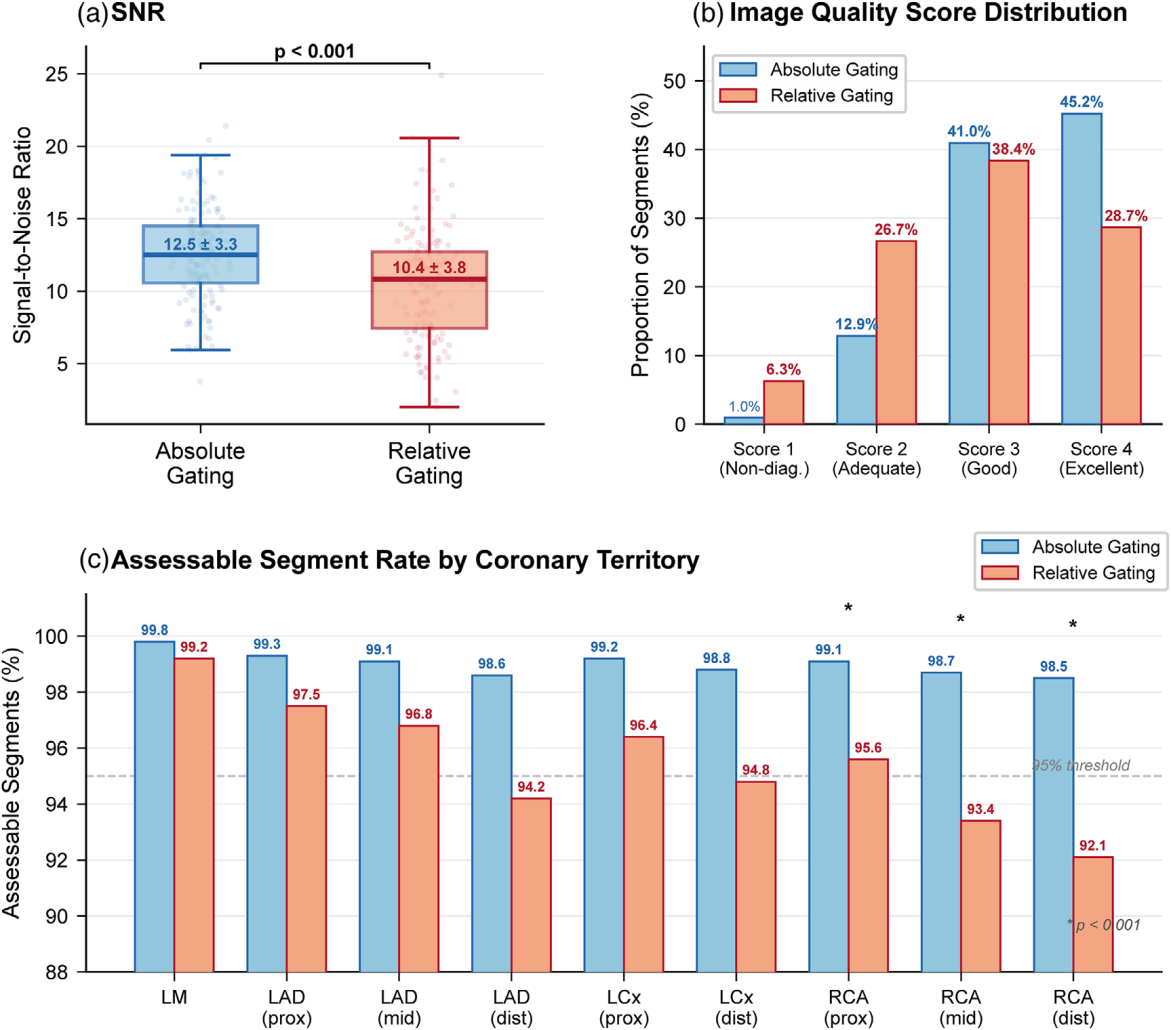
Image Quality Metrics Comparison Between Absolute and Relative Phase Gating. (a) Signal‐to‐noise ratio (SNR) box plot showing significantly higher SNR with absolute phase gating (mean 12.84 ± 3.47 vs. 10.19 ± 3.82, *p <* 0.001). Individual data points are overlaid with jittered positions. (b) Distribution of subjective image quality scores across all evaluated coronary segments (2,100 segments per group), demonstrating a higher proportion of excellent‐quality segments (score 4) and lower proportion of non‐diagnostic segments (score 1) with absolute gating. (c) Assessable segment rates by coronary territory. Absolute phase gating maintained assessability above 98% across all territories, while relative gating showed progressive decline particularly in right coronary artery (RCA) segments, which exhibit the greatest motion excursion. Dashed line indicates the 95% assessability threshold. * *p <* 0.001.

Comprehensive image quality assessment of 4,200 total coronary segments (15 segments × 280 patients) demonstrated significantly superior performance for absolute phase gating using both subjective and objective metrics (Table 4). The mean subjective image quality score was 3.21 ± 0.58 for absolute gating versus 2.84 ± 0.71 for relative gating (independent samples t‐test: *p <* 0.001, Cohen's d effect size: 0.56, indicating moderate‐to‐large clinical significance). Figure 3 presents the SNR comparison, image quality score distribution, and assessable segment rates by coronary territory. The median scores were 3 [IQR: 3–4] versus 3 [IQR: 2–3] (Mann‐Whitney U: *p <* 0.001), reflecting a shift toward higher quality grades.

**TABLE 4:** Image quality assessment and assessable segment analysis values are presented as mean ± standard deviation or percentage.

Image Quality Metric	Absolute Gating	Relative Gating
Mean IQ score	3.21 ± 0.58	2.84 ± 0.71
Assessable segments (%)	99.05	95.71
SNR	12.84 ± 3.47	10.19 ± 3.82
CNR	17.26 ± 4.53	14.32 ± 5.18
Weighted kappa	0.827	0.785

The distribution of quality scores differed significantly between techniques (chi‐square test: *p <* 0.001). Absolute gating achieved excellent quality (score 4) in 950 segments (45.24% of 2,100 segments) compared to 602 segments (28.67%) for relative gating, representing a 16.57 percentage point advantage. Good quality (score 3) was observed in 860 segments (40.95%) versus 806 segments (38.38%). Adequate but suboptimal quality (score 2) occurred in 270 segments (12.86%) versus 560 segments (26.67%), demonstrating that relative gating more frequently produced marginal but diagnostic image quality. Most critically, non‐diagnostic quality (score 1) occurred in only 20 segments (0.95%) with absolute gating versus 132 segments (6.29%) with relative gating, representing a 6.6‐fold reduction in non‐assessable segments. Motion artifacts were the predominant cause of non‐diagnostic segments, accounting for 85.0% (17/20) in the absolute gating group versus 78.8% (104/132) in the relative gating group (chi‐square: *p <* 0.001, OR: 6.93, 95% CI: 4.28–11.21).

The overall assessable segment ratio (defined as image quality score ≥2) was 99.05% (2,080 of 2,100 segments) for absolute gating versus 95.71% (2,010 of 2,100 segments) for relative gating, representing a 3.34 percentage point improvement (chi‐square: *p <* 0.001). In absolute terms, this translates to an average of 14.86 assessable segments per patient with absolute gating versus 14.36 per patient with relative gating out of the 15‐segment model. Generalized estimating equations analysis, appropriately accounting for within‐patient correlation of segment quality, confirmed that absolute gating independently predicted higher segment assessability (adjusted OR: 4.82, 95% CI: 3.14–7.39, *p <* 0.001) after controlling for heart rate (OR per 10 bpm increase: 0.78, 95% CI: 0.64–0.95, *p =* 0.014), log‐transformed calcium score (OR per unit increase: 0.82, 95% CI: 0.71–0.94, *p =* 0.005), and BMI (OR per 5 kg/m^2^ increase: 0.91, 95% CI: 0.81–1.03, *p =* 0.142).

Segment‐level analysis across coronary territories revealed that image quality improvements with absolute gating were most pronounced in the right coronary artery, which exhibits the greatest motion excursion among major epicardial vessels. For RCA segment 1 (proximal RCA), mean image quality scores were 3.28 ± 0.63 versus 2.94 ± 0.76 (*p <* 0.001). For RCA segment 2 (mid‐RCA), considered one of the most challenging segments due to anteroposterior motion, scores were 3.18 ± 0.65 versus 2.67 ± 0.84 (*p <* 0.001), representing a 0.51‐point improvement. For RCA segment 3 (distal RCA), scores were 3.08 ± 0.71 versus 2.52 ± 0.89 (*p <* 0.001). The left main artery, being relatively stationary, showed the smallest but still significant improvement: 3.54 ± 0.52 versus 3.38 ± 0.61 (*p =* 0.002). These findings support the mechanistic hypothesis that absolute gating's advantage derives from consistent capture of the systolic quiescent period when coronary motion is minimal.

Objective image quality metrics corroborated subjective assessments. Signal‐to‐noise ratio measured in disease‐free proximal coronary segments was 12.84 ± 3.47 for absolute gating versus 10.19 ± 3.82 for relative gating (*p <* 0.001), representing a 26% improvement. Contrast‐to‐noise ratio was similarly superior: 17.26 ± 4.53 versus 14.32 ± 5.18 (*p <* 0.001), a 21% improvement. Mean coronary attenuation was comparable between groups (387.54 ± 68.24 HU vs. 381.26 ± 72.18 HU, *p =* 0.284), indicating that the SNR and CNR differences reflect reduced image noise rather than altered contrast delivery. The improved SNR with absolute gating likely reflects more consistent capture of the coronary arteries during the quiescent systolic phase, resulting in reduced motion‐related blurring and more reliable signal measurement in the region of interest, rather than differences in inherent image noise between the two gating approaches.

Inter‐observer agreement for image quality assessment was excellent for both techniques, though slightly higher for absolute gating. Weighted kappa for subjective quality scores was 0.827 (95% CI: 0.798–0.856) for absolute gating versus 0.785 (95% CI: 0.751–0.819) for relative gating (z‐test for difference: *p =* 0.042), both exceeding the 0.75 threshold for excellent agreement. Intraclass correlation coefficients for objective measurements were comparably high: ICC for SNR was 0.912 (95% CI: 0.887–0.934) versus 0.894 (95% CI: 0.866–0.918) (*p =* 0.328), and ICC for CNR was 0.908 (95% CI: 0.882–0.931) versus 0.887 (95% CI: 0.858–0.912) (*p =* 0.284). The excellent inter‐observer reliability validates the robustness of image quality assessments and supports the reproducibility of these findings.

### 3.5 Subgroup analyses and effect modification

Subgroup analysis stratified by heart rate variability revealed that absolute phase gating provided incrementally greater benefit in patients with higher R‐R interval variability—precisely the population most challenging for conventional percentage‐based gating. Among patients with high heart rate variability (SD ≥24 bpm, *n =* 94 total, 33.57% of cohort), the difference in assessable segments was particularly pronounced: 98.51% (782 of 793 segments) with absolute gating versus 93.62% (744 of 794 segments) with relative gating (difference: 4.89 percentage points, chi‐square: *p <* 0.001). This represents a 5.2‐fold reduction in non‐assessable segments (1.49% vs. 6.38%). In contrast, among patients with lower heart rate variability (SD <24 bpm, *n =* 186 total), assessable segments were 99.38% (1,298 of 1,307) versus 97.09% (1,268 of 1,306) (difference: 2.29 percentage points, *p =* 0.002), representing a 3.7‐fold reduction. Formal interaction testing confirmed significant effect modification by heart rate variability category (interaction *p =* 0.018), indicating that absolute gating's advantage is magnified in patients with irregular rhythms—precisely those who would benefit most from improved image quality.

Calcium score stratification demonstrated consistent image quality improvements across the spectrum of coronary calcification burden. Even in the highest calcium score category (Agatston score >400, *n =* 73 patients), where beam hardening and blooming artifacts pose additional challenges, absolute gating maintained superior assessability: 97.83% of segments were assessable versus 93.26% for relative gating (difference: 4.57 percentage points, *p =* 0.003). In the zero calcium score category (*n =* 63), assessability was 99.68% versus 98.41% (difference: 1.27 percentage points, *p =* 0.048). In the moderate calcium category (101–400, *n =* 75), assessability was 99.11% versus 95.20% (difference: 3.91 percentage points, *p <* 0.001). Interaction testing revealed no significant effect modification by calcium score category (interaction *p =* 0.524), indicating that the image quality advantage of absolute gating persists regardless of calcification burden, though the absolute benefit is numerically largest in heavily calcified vessels where motion artifacts compound calcific blooming effects.

BMI subgroup analysis demonstrated robust performance of absolute gating across varying body habitus, as reflected in both radiation dose (discussed previously) and image quality outcomes. Among normal‐weight patients (BMI <25 kg/m^2^, *n =* 84), assessable segments were 99.52% with absolute gating versus 97.38% with relative gating (*p =* 0.004). Among overweight patients (BMI 25–30 kg/m^2^, *n =* 114), assessability was 99.06% versus 96.14% (*p <* 0.001). Among obese patients (BMI >30 kg/m^2^, *n =* 82), assessability was 98.54% versus 93.58% (*p <* 0.001). The slightly lower absolute assessability in obese patients reflects increased image noise from higher x‐ray attenuation, but the relative advantage of absolute gating was maintained across all BMI categories (interaction *p =* 0.671). Mean image quality scores showed similar patterns, with absolute gating achieving scores of 3.28 ± 0.53, 3.19 ± 0.58, and 3.14 ± 0.62 in the three BMI categories respectively, compared to 2.91 ± 0.68, 2.82 ± 0.71, and 2.76 ± 0.74 for relative gating.

## 4 DISCUSSION

This retrospective matched cohort study demonstrates that absolute phase gating was associated with a substantially lower radiation dose (approximately 64%) while maintaining or improving diagnostic accuracy and image quality in patients with AF undergoing CCTA. The reduction in median effective dose from 7.35 mSv to 2.63 mSv without compromising diagnostic performance is consistent with the goals of the Image Wisely initiative for dose‐optimized cardiac imaging.

The dose reduction, while anticipated given the inherently shorter systolic acquisition window and the predominant use of single‐beat high‐pitch acquisition in the absolute gating group compared with the wider and more frequently retrospective windows used for relative gating, aligns with recent literature on cardiac CT dose optimization. The PROTECTION VI registry reported a median DLP of 195 mGy·cm for contemporary CCTA protocols.^19^ Our absolute gating cohort achieved an even lower median DLP (187.50 mGy·cm), positioning this technique among the most dose‐efficient approaches. Previous studies comparing prospective versus retrospective gating in patients with AF reported dose reductions of similar magnitude.^20^, ^21^ It should be emphasized, however, that this dose advantage reflects the acquisition strategy that absolute gating renders reliably feasible in irregular rhythms—chiefly a narrow systolic window amenable to high‐pitch triggering—rather than the fixed‐delay timing per se; a relative gating protocol confined to a comparably narrow window with matched dose modulation would be expected to narrow this difference. The two protocols also differed in padding‐window design—a fixed 100‐ms window for the absolute slow‐helical acquisitions versus a variable 15% R‐R window for relative gating—which may contribute to the observed differences independent of the fixed‐delay principle.

The diagnostic accuracy observed in our study compares favorably with meta‐analyses of CCTA performance. Our findings of 96.61% sensitivity and 96.97% NPV with absolute gating confirm that dose optimization does not compromise diagnostic utility.^22^ The comparable accuracy between absolute and relative gating groups demonstrates that technical modifications for AF do not necessitate trade‐offs between dose and diagnostic performance.

The superior performance of absolute phase gating in patients with AF can be attributed to fundamental physiological principles. Cardiac systole duration remains relatively constant (280–350 milliseconds) across heart rates, whereas diastole is highly variable.^23^ In AF, percentage‐based gating targets inconsistent cardiac phases, while absolute phase gating circumvents this by consistently capturing the mid‐systolic quiescent phase regardless of R‐R variability.^24^

Several limitations warrant consideration. The retrospective single‐center design may limit generalizability. Only 42.14% of patients underwent invasive angiography for diagnostic accuracy assessment, potentially introducing verification bias. The study employed a single CT scanner platform, and results may vary with different manufacturers. Additionally, effective dose estimation using a single conversion factor (*k* = 0.014 mSv·mGy^−^^1^·cm^−^^1^) does not account for patient‐specific factors including body size, age, and sex; therefore, CTDIvol and DLP should be considered the primary dose metrics in this study. The repeat‐acquisition rates were low in both groups (2.86% with absolute and 1.43% with relative gating; *p =* 0.683). These low rates likely reflect whole‐heart coverage acquired within a single breath‐hold—predominantly single‐beat high‐pitch acquisition in the absolute gating group—together with the prospective exclusion of patients with frequent (>10%) premature atrial or ventricular contractions and of technically inadequate studies; repeat rates may be higher with multi‐beat acquisition strategies or in less selected populations. Future prospective multicenter trials with standardized protocols would provide higher‐level evidence.^25^

## 5 CONCLUSIONS

In patients with atrial fibrillation undergoing coronary CT angiography, absolute phase gating can potentially reduce radiation dose while maintaining or improving diagnostic accuracy and image quality. In our cohort it achieved a median effective dose of 2.63 mSv with nearly complete segment assessability. Because part of the observed dose reduction is attributable to the shorter systolic acquisition window and the predominant use of high‐pitch acquisition rather than to the fixed‐delay timing itself, the magnitude of benefit will depend on the comparator protocol. Absolute phase gating nonetheless represents an attractive dose‐optimized option in this challenging population and warrants prospective, multivendor confirmation.

## AUTHOR CONTRIBUTIONS

**Yuyang Li**: Conceptualization; data curation; formal analysis; investigation; methodology; resources; validation; writing—original draft; writing—review and editing.

## CONFLICT OF INTEREST STATEMENT

The authors declare no conflicts of interest.

## ETHICS STATEMENT

This study was approved by the Ethics Committee of Wuhan Fourth Hospital (Approval No. KY2025‐201‐21), with waiver of informed consent granted due to the retrospective design and minimal risk to participants. All methods were carried out in accordance with Declaration of Helsinki.

## Data Availability

The data supporting the findings of this study are available from the corresponding author upon reasonable request.
